# Bone Health Improvement Protocol

**Published:** 2017-08-30

**Authors:** Nathan K. Wool, Shannon Wilson, Alexander CM Chong, Bradley R. Dart

**Affiliations:** 1 University of Kansas School of Medicine-Wichita, Department of Orthopaedics; 2 Via Christi Health, Wichita, KS

**Keywords:** metabolic bone disease, bone fractures, osteoporosis, secondary prevention

## Abstract

**Introduction:**

Metabolic bone disease is a malady that causes significant morbidity and mortality to a patient who has sustained a fragility fracture. There is currently no protocol to prevent secondary fragility fracture at our institution. The objective of this study was to create an appropriate protocol for implementing clinical pathways for physicians to diagnose and treat osteoporosis and fragility fractures by educating patients.

**Methods:**

A multidisciplinary team created an appropriate protocol that could be implemented in an inpatient setting. A thorough literature review was conducted to evaluate potential barriers and efficacious methods of protocol design.

**Results:**

A bone health improvement protocol was developed. Any patient over the age of 50 who sustains a fracture from low energy trauma, such as a fall from standing or less, should be considered to place into this protocol. These patients received education on metabolic bone disease, a prescription for high dose vitamin D therapy, and laboratory testing to determine the etiology of their metabolic bone disease. Continuity of care of these patients with their primary care provider was provided for further management of their metabolic bone disease and evaluation of their disease after discharged from the hospital.

**Discussion:**

Comprehensive secondary prevention should consist of osteoporosis assessment and treatment together with a fall risk assessment. With this protocol, secondary fragility fractures potentially could be prevented.

## Introduction

Osteoporosis is a prevalent metabolic bone disease among the elderly. It is defined as a disorder with micro-architectural deterioration that impairs both bone structural properties and bone quality.^1^ This is a significant public health issue that predisposes 50% of patients over age of 50 year-old to an increased risk for fragility fracture.^1^–^5^ Fragility fractures, defined as bone fractures resulting from a low-energy trauma such as fall from a standing height or less, are a consequence of low bone quality and density.^6^,^7^ These types of fractures are encountered most commonly in the hip, spine, distal radius, and proximal humerus. Hip fractures are the major cause of morbidity and mortality associated with osteoporosis and fragility fractures.^1^ The risk of mortality for elders after a hip fracture due to low energy trauma is twice that of the general population. Osteoporosis and fragility fractures pose enormous challenges for both the individual and society in terms of loss of independence, quality of life, and economic burden.^8^,^9^ These include long hospitalization, need for surgical treatment, increased disability, and partial or complete loss of the ability to perform activities of daily living independently.

El-Rabbany et al.^10^ found only 5% to 38% of patients with fragility fractures were being treated for osteoporosis at final follow-up. Identifying patients at risk and getting the proper evaluation and treatment is not universal. A disconnect exists between the realization that fragility fractures are the stigmata of osteoporosis and the engagement of the patient and physician team toward more universal diagnosis and treatment.

Orthopedic residency and curricula may not provide sufficient knowledge or training to allow osteoporosis management.^11^ Edwards et al.^12^ demonstrated a modest increase in certain aspects of bone health order pathways and treatment by using an electronic medical record order set. That intervention created with providers’ input increased the follow-up and treatment of patients with osteoporosis. This, however, failed to increase diagnosis or treatment for osteoporosis at the time of hospitalization for a fragility fracture.

No protocol exists to prevent secondary fragility fracture at our institution. The objective of this study was to create an appropriate protocol for implementing clinical pathways for physicians to diagnose and treat osteoporosis and fragility fractures by educating patients.

## Methods

A multidisciplinary team, which consisted of physicians, nurse practitioners, physical therapists, nurse managers, and the orthopedic service line coordinator, was assembled to create an appropriate protocol that could be implemented at a level 1 trauma center about metabolic bone disease and fragility fractures. This team was tasked with vetting the protocol to ensure feasibility in implementation at a clinical level as well as ease of modification after implementation. A thorough literature review was conducted to evaluate efficacious methods of protocol design and potential barriers to implementation. The literature reviews also encompassed treatment goals for patients with osteoporosis and fragility fracture.

## Results

**Figure 1:**
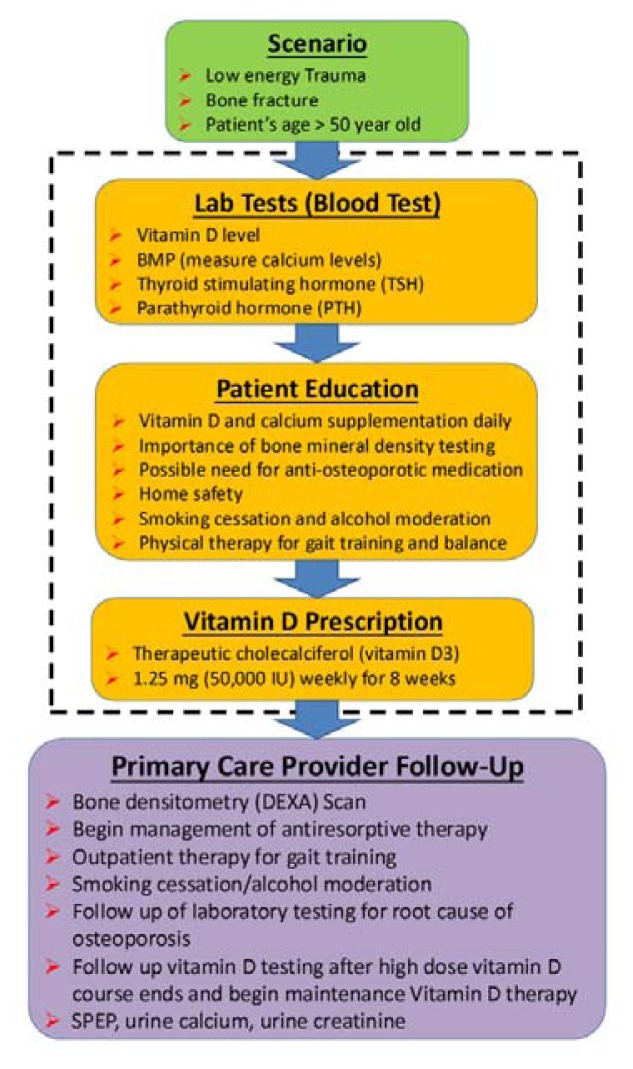
The bone health protocol with treatment goals to be provided before the patient leaves the hospital.

A bone health protocol was developed by the multidisciplinary team (Figure 1). This protocol was created to improve care for patients at risk for fragility fracture or post-fracture which focused on initiating and facilitating the screen and treatment of osteoporosis. It divided into several strategic steps: scenario, lab tests, patient education, vitamin D prescription, and primary care provider follow-up.

## Scenario

Orthopedic surgeons, trauma surgeons, and emergency room (ER) providers often are the first clinicians to see the patients after a fragility fracture. A patient over the age of 50 years who sustains a fracture to the appendicular skeleton caused by a low energy trauma event, such as a fall from standing height or less, should be placed into this protocol. The incidence and lifetime risk of any fracture doubles every decade after 50 year-old.^4^,^11^,^13^–^15^ During the visit, a standard medical history also should be obtained, with particular attention paid to age, weight, personal and family history of fracture, physical inactivity, medication, alcohol, tobacco, and frailty.

## Laboratory Tests (Blood Test)

Although bone strength cannot be determined directly in vivo, with increased age comes a marked reduction in bone mass and destruction of bone architecture, leading to a considerable decrease in bone strength.^16^–^18^ Basic laboratory tests such as a complete blood count, serum chemistry profile, and urinalysis usually requested by the clinicians during a regular medical checkup as part of a routine checkup to monitor patient health, and these tests would be a good begin determining the ethology for the metabolic bone disease by including a basic metabolic profile, parathyroid hormone level, thyroid stimulating hormone, follicle-stimulating hormone level, luteinizing hormone level, and Vitamin D-25-hydroxy level. The results of these tests give clinicians insight into patient’s status regarding common etiologies of metabolic bone disease including decreased vitamin D levels, hyperparathyroidism, hyperthyroidism, renal osteodystrophy, and hypogonadism. These tests are among the most effective in determining etiology of metabolic bone disease based on literature.^19^

## Patient Education

Patients with osteoporosis who have sustained a fracture have a very high risk of suffering a new fracture, often within 1 year of original fracture.^14^,^20^–^28^ Therefore, before discharge the patients from the hospital, nursing staff, and healthcare providers must provide proper education to patients and their family members about metabolic bone disease. This includes acute management of the presenting fracture and prevention of secondary fragility fractures. The education shall include the importance of vitamin D and calcium supplementation, bone mineral density (BMD) testing, the possible need for anti-osteoporotic medications, home safety goals, smoking cessation and alcohol moderation, and physical therapy for gait training and balance. Patients who underwent balance training and education had better balance measures and fear of falling outcomes.^29^–^31^ Smokers should be advised to quit, patients with alcoholism should be treated, and patients for whom risk factor analysis indicates a strong potential for osteoporosis should have an ultrasound of the heel as an initial screening tool for every 6 months followed by a DEXA test in those identified as having low bone density.^32^ This education will allow patients and their family members to be involved in the patient’s bone health.

## Vitamin D Prescription

Vitamin D is essential for normal calcium metabolism and maintenance of bone density, and the risk of deficiency increases with age.^33^–^36^ The prevalence of vitamin D deficiency in 2010 in the US was 41.6% with deficiency rates around 49% in patients age of 55 to 64 year-old.^37^ Due to the high levels of vitamin D deficiency among elderly individuals and the delay in test results which may take up to 5 days due to the specialized nature of the test and scarcity of laboratories that perform it, there is a benefit to start therapeutic cholecalciferol (vitamin D3) prophylactically before discharge from the hospital. Supplementation with vitamin D reduced bone loss and the incidence of non-vertebral fractures in men and women aged ≥65 years old.^38^ Patients should be prescribed vitamin D supplementation (vitamin D3) for 8 weeks at a dose of 1.25 mg or 50,000 international units (IU) once a week which is supported by previous studies.^39^–^41^ With this high dose of vitamin D supplementation (50,000 IU) cholecalciferol restored serum 25-hydroxyvitamin D (25(OH)D) levels to sufficient levels i.e. above vitamin D deficiency level (50 nmol/L)^42^,^43^ among migrants and non-migrants especially for those with lower baseline serum 25(OH)D.

## Primary Care Provider Follow-up

The ultimate goal in treating fragility fracture patients with osteoporosis is not only acute management of the presenting fracture, but also the prevention of subsequent fractures.^7^,^44^–^47^ The primary care providers are the crucial members that need to provide continuity of care with these patients on their metabolic bone disease management and further evaluation of their disease after the patient is discharged from the hospital. A bone densitometry (DEXA) scan should be scheduled to measure and evaluate BMD. The results of the DEXA scan can be used to gauge the severity of bone loss, predict future fracture risk, make treatment decisions, and monitor changes in BMD related to age, medical conditions, or therapeutic intervention. The provider should discuss initiation of antiresorptive therapy, outpatient physical therapy for gait training, smoking cessation, and alcohol intake moderation.

By evaluating the initial routine laboratory test results, the primary care providers should make the decision on the continuing therapeutic cholecalciferol (vitamin D3) treatment and, if necessary, order further testing such as serum protein electrophoresis, 24-hour urine calcium, and 24-hour urine creatinine to delineate the etiology of the metabolic bone disease.

## Discussion

The goal of this bone health improvement protocol was to ensure that patients with osteoporosis and fragility fractures receive quality care for their bone health in both the inpatient and outpatient settings. About 10 million Americans over the age of 50 have osteoporosis and that number will increase to 14 million by 2020.^15^ These patients are high risk and should be monitored closely for osteoporosis in the setting of any fracture. There is a large gap between what has been learned and what is applied by patients and health care providers. The biggest problem is a lack of awareness of bone disease among both the public and health care professionals.^32^

Patients who have had a fragility fracture have hypovitaminosis D 73% of the time.^48^ This is in line with current levels in the United States and Canada in a population without fracture. A prospective randomized trial in 2009 demonstrated a trend toward a decrease in fracture incidence in patients who took daily vitamin D and calcium supplementation.^49^ High dose vitamin D3 (≥ 300,000 IU) is efficacious in treating low levels of vitamin D and restoring the level to a normal limit.^39^ Kearns et al.^50^, however, concluded that a high dose of vitamin D3 (≥ 600,000 IU) at one time will have adverse effects which could cause hypercalcemia or hypercalcinuria.

Osteoporosis is thought to be caused by factors including age-related impairment of bone formation, decreased calcium and vitamin D intake, decreased physical activity, and estrogen’s positive effects on calcium balance in the intestines, kidneys, and bone.^51^ Providing patients with the adequate information to take control of their osteoporosis is crucial to the success of this protocol. Patient education such as the benefit of smoking cessation should be emphasized, especially in the setting of fracture. Tobacco has been shown to hinder fracture healing and alcohol consumption of three or more units per day will have consequential effects on bone health, leading to lower BMD when compared with more moderate drinking.^6^ Education on avoidance of preventable falls also has a major impact on reducing further fragility fractures as patients with osteoporosis often experience muscle weakness, postural deformity, and poor balance.^52^ Patients who undergo tailored exercises and intervention have a decrease in fall rate in the community.^53^ All these measures and education should assist in decrease the rate of recurrent fragility fracture.

Elderly patients presenting with fragility fractures should be offered assessment with this protocol by orthopedic and/or trauma surgeon teams, as they have a unique opportunity to diagnose, arrange follow-up, and ensure the patient is started on the appropriate therapy. The orthopedic and trauma surgeons should communicate clearly with the primary care physicians the need to explore and address the relevant causes.

Genetics and nutrition contribute to the rapid phase of bone loss in postmenopausal women and the slow phase of bone loss in aging women and men.^32^ These factors appear to be largely the result of estrogen deficiency. Estrogen is a hormone that is important throughout life to bone development and maintenance in both men and women. Drugs, such as antiresorptives, that prevent bone breakdown have been effective in reducing the risk of future fractures. These drugs not only slow any further deterioration of the skeleton, but also allow for some repair and restoration of bone mass and strength. However, cannot completely restore mechanical integrity because of the absence of an anabolic effect.

There are several possible barriers to implementation of this protocol in the current healthcare setting. The most notable is provider education. Healthcare providers of fragility fracture patients will need to be well educated on the protocol for it to be effective. These healthcare providers include orthopedic physicians, emergency room physicians, emergency room physician extenders, orthopedic mid-level providers, primary care providers, resident physicians, and nurses. After adequate education, the protocol implementation could be subjected to improvements that would incorporate potential patients into all aspects of the protocol. Another potential barrier was identified through a survey of physicians which was cost of the workup necessary for osteoporosis.^54^ Communication between orthopedic surgeons and primary care physicians also has been identified as a barrier that needs to be addressed.^55^ Increasing awareness of the responsibility and the opportunity providers have to make a substantial impact on this clinical problem would prevent secondary fragility fractures and decrease the morbidity and mortality of patients with underlying metabolic bone disease. Other potential barriers include cost of therapy, patient reluctance, time and cost of diagnosing osteoporosis, special patient populations (e.g., uninsured or underinsured, minorities) gender differences, lack of access to BMD testing, and a lack of time to address secondary prevention.^56^–^57^

Comprehensive secondary prevention should consist of osteoporosis assessment and treatment together with a fall risk assessment. With this protocol, secondary fragility fractures could be prevented.
